# Novel Variants of Streptococcus thermophilus Bacteriophages Are Indicative of Genetic Recombination among Phages from Different Bacterial Species

**DOI:** 10.1128/AEM.02748-16

**Published:** 2017-02-15

**Authors:** Paula Szymczak, Thomas Janzen, Ana Rute Neves, Witold Kot, Lars H. Hansen, René Lametsch, Horst Neve, Charles M. A. P. Franz, Finn K. Vogensen

**Affiliations:** a Bacterial Physiology and Improvement, R&D Microbial Platform, Chr. Hansen A/S, Hørsholm, Denmark; b Department of Food Science, University of Copenhagen, Frederiksberg, Denmark; c Department of Environmental Science, Aarhus University, Roskilde, Denmark; d Department of Microbiology and Biotechnology, Max Rubner-Institut, Kiel, Germany; The Pennsylvania State University

**Keywords:** bacteriophages, classification, Streptococcus thermophilus, structural genes

## Abstract

Bacteriophages are the main cause of fermentation failures in dairy plants. The majority of Streptococcus thermophilus phages can be divided into either *cos*- or *pac*-type phages and are additionally characterized by examining the V2 region of their antireceptors. We screened a large number of S. thermophilus phages from the Chr. Hansen A/S collection, using PCR specific for the *cos*- or *pac*-type phages, as well as for the V2 antireceptor region. Three phages did not produce positive results with the assays. Analysis of phage morphologies indicated that two of these phages, CHPC577 and CHPC926, had shorter tails than the traditional S. thermophilus phages. The third phage, CHPC1151, had a tail size similar to those of the *cos*- or *pac*-type phages, but it displayed a different baseplate structure. Sequencing analysis revealed the genetic similarity of CHPC577 and CHPC926 with a subgroup of Lactococcus lactis P335 phages. Phage CHPC1151 was closely related to the atypical S. thermophilus phage 5093, homologous with a nondairy streptococcal prophage. By testing adsorption of the related streptococcal and lactococcal phages to the surface of S. thermophilus and L. lactis strains, we revealed the possibility of cross-interactions. Our data indicated that the use of S. thermophilus together with L. lactis, extensively applied for dairy fermentations, triggered the recombination between phages infecting different bacterial species. A notable diversity among S. thermophilus phage populations requires that a new classification of the group be proposed.

**IMPORTANCE** Streptococcus thermophilus is a component of thermophilic starter cultures commonly used for cheese and yogurt production. Characterizing streptococcal phages, understanding their genetic relationships, and studying their interactions with various hosts are the necessary steps for preventing and controlling phage attacks that occur during dairy fermentations.

## INTRODUCTION

Bacteriophages represent a major threat to the dairy industry. Phage infections of starter cultures cause acidification delays or failures, frequently leading to lower-quality cheese and fermented dairy products (1). Therefore, developing efficient strategies to prevent and combat phage attacks is mandatory in large-scale dairy fermentations. To that end, accurate characterization and grouping of dairy phages, as well as better detection methods and understanding of phage evolution, are required.

Streptococcus thermophilus phages are especially important due to the increased commercial use of thermophilic starter cultures, which are selected bacterial strains used for the manufacture of fermented dairy foods (2). Almost all known S. thermophilus phages show similar overall characteristics, displaying double-stranded DNA (genome size, 30 kb to 45 kb) packed into an isometric capsid (45 to 60 nm in diameter) connected to a long noncontractile tail (200 to 260 nm in length). Hence, they belong to the Siphoviridae family of the Caudovirales order (2, 3). In addition to their morphological similarity, many S. thermophilus phages seem to be homologous on a genetic level (above 80% nucleotide similarities over 50% query cover). Even phages from distant geographic origins share the same genome organization (2, 4).

Over time, researchers have proposed methods for classifying S. thermophilus phages. The most used grouping distinguishes two subgroups, the *cos*- or *pac*-type phages, correlating the DNA packaging mechanism with a set of two or three major structural proteins, respectively (5). Another important grouping is based on the variable region VR2 of the antireceptor gene of S. thermophilus phages. This method enables linking of the detected phage with its host specificity (6).

The discovery of a phage with novel properties, called 5093 (7), challenged the current classification system. This atypical S. thermophilus phage is not a standard *cos*- or *pac*-type phage representative, and it has no definite antireceptor gene recognized in its genome. It shares more nucleotide similarity with a nondairy streptococcal prophage rather than dairy phage species. Moreover, phage 5093 exhibits unusual morphological features, i.e., globular structures on the phage tip, that have not been observed in other S. thermophilus phages (7). To date, phage 5093 is the only reported representative with this unique characteristic (8).

Analysis of the sequencing data of various S. thermophilus phages enabled the investigation of putative evolutionary mechanisms for the group. Some researchers postulated that horizontal gene transfer and exchange of genetic modules between various phage species could be the major mechanisms for phage development (7, 9, 10). This observation is highly important in relation to dairy phages. The recombination process may result in extending their host range and impede the successful control of phage infections on starter cultures, which frequently consist of mixtures of various lactic acid bacteria (2, 11).

The results of this study have brought more insights into the characteristics and the evolution of S. thermophilus phages. By sequencing and analyzing the genomes of 59 S. thermophilus phages from the Chr. Hansen A/S collection, we discovered three atypical representatives that could not be classified according to the known groupings. The first unusual phage identified seemed to be highly related to the novel phage 5093. The two others shared parts of their genomes with phages infecting Lactococcus lactis, a bacterium commonly used for mesophilic dairy fermentations. By studying the adsorption of closely related lactococcal and streptococcal phages to selected strains of S. thermophilus and L. lactis, the interactions of phages with nonpermissive hosts were revealed. The results of this study showed that S. thermophilus phages are likely to inherit structural genetic modules from various phages of dairy and nondairy species. The results highlighted the necessity of considering ongoing phage evolution when studying phage-host interactions in dairy fermentations. A notable diversity among S. thermophilus phage populations requires that a new classification of the group be proposed.

## RESULTS

### Characterization of the Chr. Hansen A/S phage collection.

Chr. Hansen A/S possesses a large collection of Streptococcus thermophilus phages isolated from dairy fermentations. We tested 59 phages from the collection with the *pac*- and *cos*-grouping multiplex PCR method (12). As a result, 37 phages could be classified as *cos* type and 19 phages as *pac* type; however, three phages gave negative results with the assay. Moreover, the region VR2, which is characteristic of the majority of S. thermophilus phages, was not detected, as no bands were visible in an additional PCR (6) performed for the three phage genomes. The three phages, called CHPC577, CHPC926, and CHPC1151, were therefore expected to be atypical and were selected for a more detailed investigation.

### Morphological features of the atypical phages.

**FIG 1:**
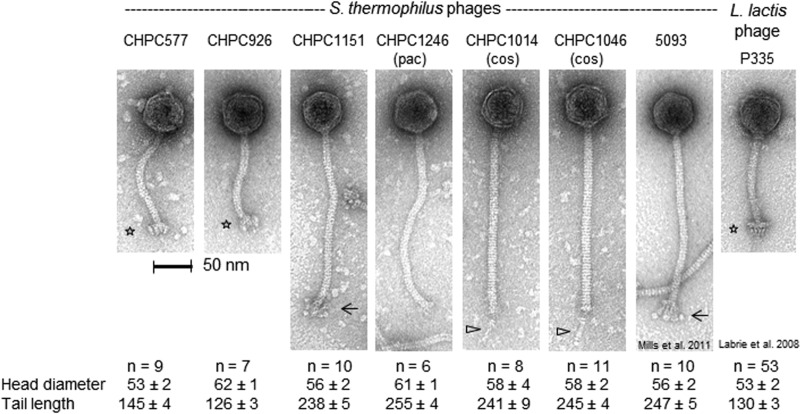
Transmission electron micrograph images of the atypical S. thermophilus phages CHPC577, CHPC926, and CHPC1151, of the *pac*-type phage CHPC1246, and of the *cos*-type phages CHPC1014 and CHPC1046. The previously described S. thermophilus phage 5093 (described in reference 7) and the L. lactis phage P335 (described in reference 13) are also shown on the right side. Phages were negatively stained with 1% uranyl acetate. The symbols indicate distinct baseplate structures (asterisks), flexible globular baseplate appendices (arrows), and single central tail fibers (triangles). Head and tail sizes are indicated in nanometers.

The electron microscopic analysis of the three studied phages revealed morphotypes that were unusual for typical S. thermophilus phages (2). Phage CHPC577 had a head of 53 nm in diameter and a tail length of 145 nm. Phage CHPC926 revealed an isometric head of 62 nm in diameter and a tail of 126 nm in length. Both phages had much shorter tails than those of typical S. thermophilus phages. Moreover, they possessed unusual baseplate structures on the tail tips. The morphological features of CHPC577 and CHPC926 resembled L. lactis phage P335 (13). The third phage studied, CHPC1151, revealed a similarity to the novel S. thermophilus phage 5093. A head size of 56 nm, a tail length of 238 nm, and globular fluffy baseplates were comparable in both phages (Fig. 1).

### Genomic features of the atypical phages.

We analyzed the complete nucleotide sequences for phages CHPC577, CHPC926, and CHPC1151. The studied phages had genome lengths of 35,129 bp, 30,077 bp, and 33,507 bp, respectively, as well as mol% G+C contents of 36.8%, 37%, and 38.5%, respectively. Using RASTtk (14), 50 open reading frames (ORFs) were annotated for phage CHPC577, and one of them, ORF 28, was located on the opposite strand. Phages CHPC926 and CHPC1151 had 44 and 46 ORFs, respectively, and they were all located on the same strand. Several overlapping ORFs were detected in the genomes. Start codons for the majority of the annotated ORFs began with the ATG sequence. Other variants of start codons are listed in Table S1. The absence of a complete lysogeny module, providing the integration of a phage genome into a bacterial chromosome, suggested that phages CHPC577, CHPC926, and CHPC1151 were lytic phages and could not modify into a prophage stage. The three investigated phages exhibited a modular genetic architecture consisting of late-, early-, and middle-expressed regions common to the Siphoviridae family (10). A summary of the phages' characteristics is presented in Table 1.

**TABLE 1:** Characteristics of S. thermophilus phages CHPC577, CHPC926, and CHPC1151

Characteristic	CHPC577	CHPC926	CHPC1151
Bacterial host strain	CHCC2389	CHCC2138	CHCC9743
Origin	Cheese plant in Italy	Yogurt plant in France	Cheese plant in Italy
Life cycle	Virulent	Virulent	Virulent
Length (bp)	35,129	30,077	33,507
G+C content (%)	36.8	37	38.5
Predicted ORFs (no.)	50	44	46

**FIG 2:**
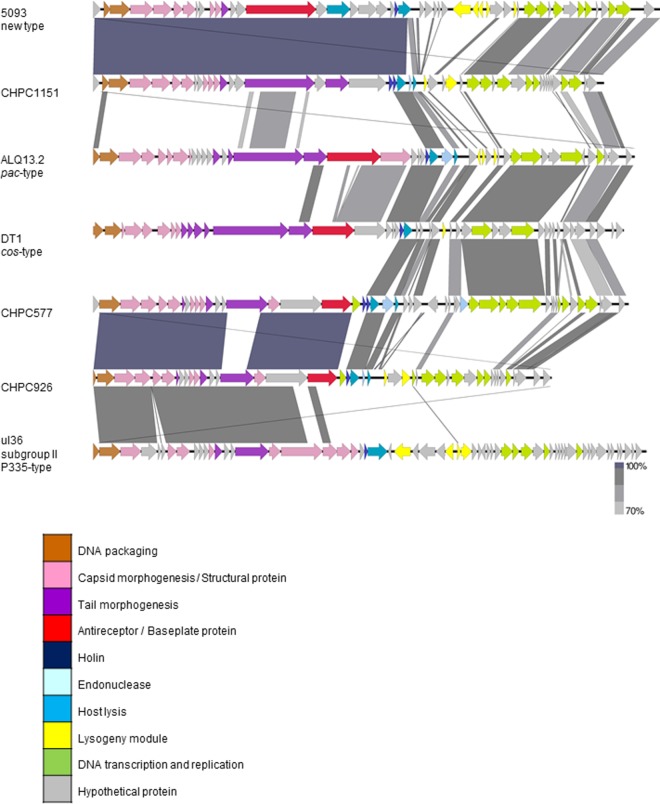
Schematic comparison of nucleotide sequences of phages CHPC577, CHPC926, and CHPC1151 with nucleotide sequences of phages 5093 (accession no. FJ965538), ALQ13.2 (accession no. FJ226752), DT1 (accession no. AF085222), and ul36 (accession no. AF349457).

A genome homology examination, based on comparing protein sequences with the translated nucleotide database available in GenBank (http://www.ncbi.nlm.nih.gov/GenBank/), suggested that phages CHPC577, CHPC926, and CHPC1151 were only partially related to typical S. thermophilus phages. As for phage CHPC577, the module coding for structural proteins was 86% similar to the Lactococcus lactis phage ul36 (accession no. AF349457). The regions covering replication and host cell lysis were 84 to 86% similar to the *cos*-type S. thermophilus phage DT1 (accession no. AF085222) and the *pac*-type S. thermophilus phage ALQ13.2 (accession no. FJ226752). Phage CHPC926 also showed genetic similarity with the L. lactis phage ul36. Nearly 50% of the CHPC926 genome was 78% alike to this species. Although both phages CHPC577 and CHPC926 revealed high genetic similarity with the same L. lactis phage, they originated from different dairy environments in various locations in Europe (Table 1). The great majority (87%) of the sequence of the third investigated phage, CHPC1151, revealed 93% similarity to the novel S. thermophilus phage 5093 (accession no. FJ965538) (7). Phage CHPC1151 originated from an Italian cheese plant, whereas phage 5093 was isolated from a mozzarella whey sample in The Netherlands (accession no. FJ965538). The nucleotide sequence similarities of the three studied phages, streptococcal phages 5093, ALQ13.2, and DT1, and lactococcal phage ul36 were visualized using Easyfig (15) and are presented in Fig. 2.

In order to verify the homology of each gene annotated for phages CHPC577, CHPC926, and CHPC1151, the highest BLAST hits of translated ORFs with the organisms in GenBank were investigated. The structural and DNA packaging genes of phages CHPC577 and CHPC926, in ORFs 1 to 20 and ORFs 1 to 18, respectively, were related to phage ul36, ul36.k1, TP901-1, Tuc2009, or P335, all belonging to subgroup II of L. lactis P335 phages (16). The corresponding module of phage CHPC1151, in ORFs 1 to 20, was homologous to the novel S. thermophilus phage 5093. For all three phages, proteins involved in the process of lysis, replication, and transcription originated from various S. thermophilus phages reported before June 2016 (Tables S2 to S4).

**FIG 3:**
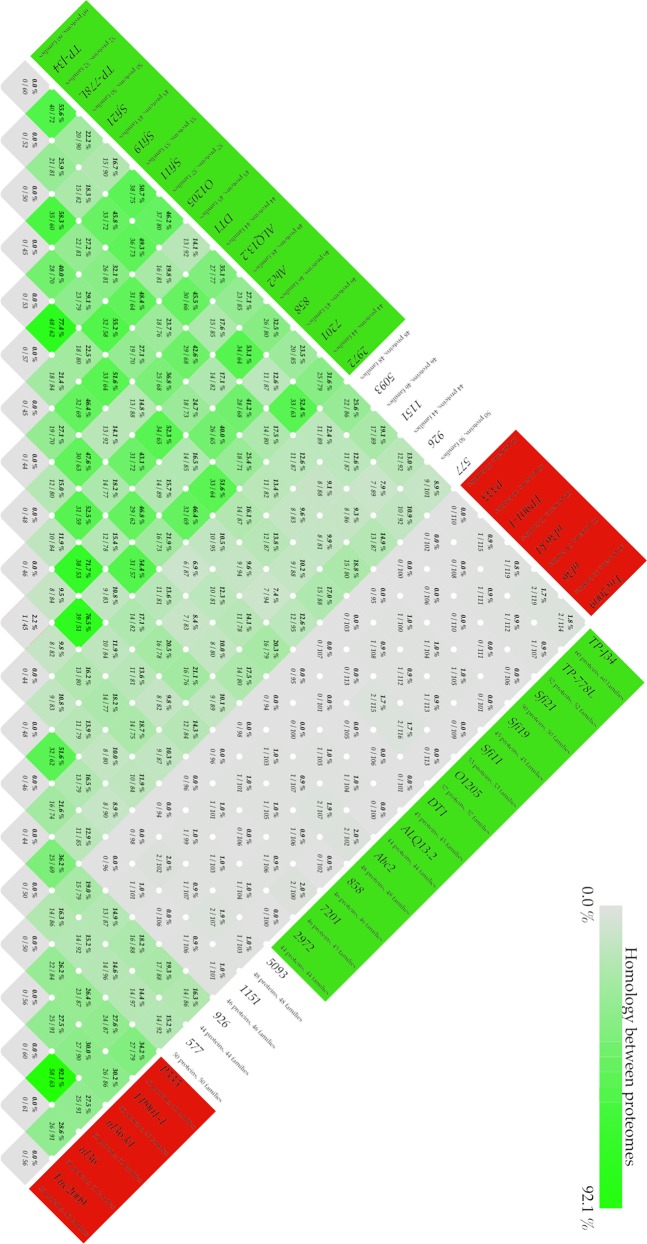
All-against-all comparison of phage proteins using BLAST. A BLAST hit is considered significant if 50% of the alignment consists of identical matches and the length of the alignment is 50% of the largest gene. Groups are marked in different colors: red, subgroup II L. lactis P335 phages; white, atypical S. thermophilus phages CHPC577, CHPC926, CHPC1151, and 5093; green, typical *cos*-type and *pac*-type S. thermophilus phages available in GenBank.

The protein homologies of the three atypical phages together with 13 S. thermophilus phages and five subgroup II L. lactis P335 phages available in GenBank were investigated. An all-against-all BLAST comparison of the phage proteins led to the identification of core genomes for specific phage types (Fig. 3). All analyzed S. thermophilus phages shared at least 7% protein homology. There were three genes conserved for the S. thermophilus phages but none of the subgroup II L. lactis P335 phages. These genes coded for lysin, DNA-binding protein, and proteins of unknown function (Tables S2 to S4).

Phages CHPC577 and CHPC926 were the only S. thermophilus phages that showed notable homology with subgroup II of L. lactis P335 phages. They held up to 20% similarity with the proteins found in the lactococcal phages. For the other S. thermophilus phages, the homology with the L. lactis phage subgroup was below 2% (Fig. 3). Lactococcal phages held 16 core genes, and 12 of these genes belong to the core genomes of CHPC577 and CHPC926 (Tables S2 to S4). Additionally, phages CHPC577 and CHPC926 held two conserved genes, ORF 7 (major tail protein) and ORF 18 (upper baseplate protein), that were unique for them. Phage CHPC1151 and the novel phage 5093 shared over 50% protein homology (Fig. 3). They held a core genome with 16 genes specific for them. The detected genes belonged to the late-expressed regions and coded for structural features, large terminase, and some unknown proteins (Tables S2 to S4).

**FIG 4:**
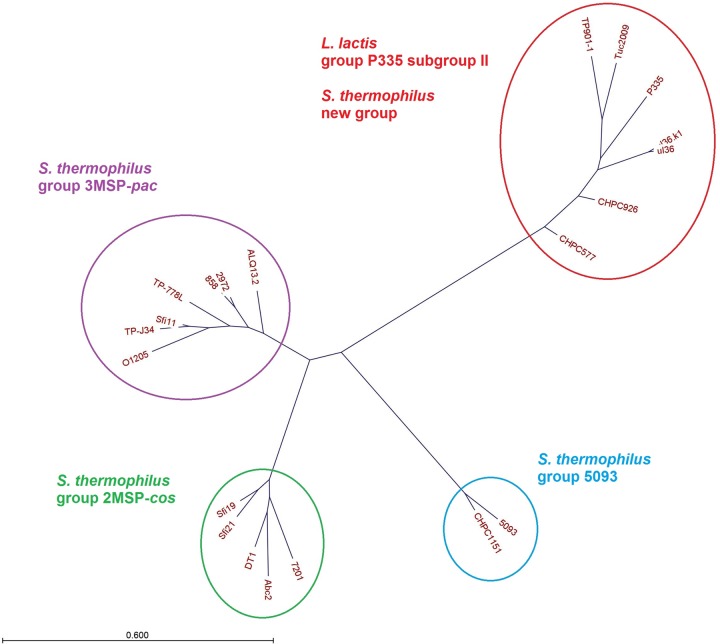
Phylogenetic tree of phages CHPC577, CHPC926, and CHPC1151 aligned with the S. thermophilus phages and the subgroup II L. lactis P335 phages available in GenBank, based on nucleotide sequences (MSP, major structural proteins).

In order to evaluate the phylogenetic relationship between phages, a multiple alignment of the nucleotide sequences from phages CHPC577, CHPC926, and CHPC1151, as well as 13 S. thermophilus phage genomes and five L. lactis phage genomes from the subgroup II P355 available in GenBank, was performed. Different gene organizations were visible for typical S. thermophilus *cos*-type phages, typical S. thermophilus *pac*-type phages, atypical phages CHPC1151 and 5093, as well as CHPC577, CHPC926, and lactococcal phages (Fig. S1). A nucleotide comparison resulted in a phylogenetic tree dividing phages into four clusters: S. thermophilus *cos* with two main structural proteins, S. thermophilus *pac* with three main structural proteins, S. thermophilus 5093, and L. lactis P335 subgroup II. Phage CHPC1151 belonged to the 5093 type, while phages CHPC577 and CHPC926 clustered together with the subgroup II of L. lactis P335 phages (Fig. 4).

### Structural proteins of the atypical phages.

**FIG 5:**
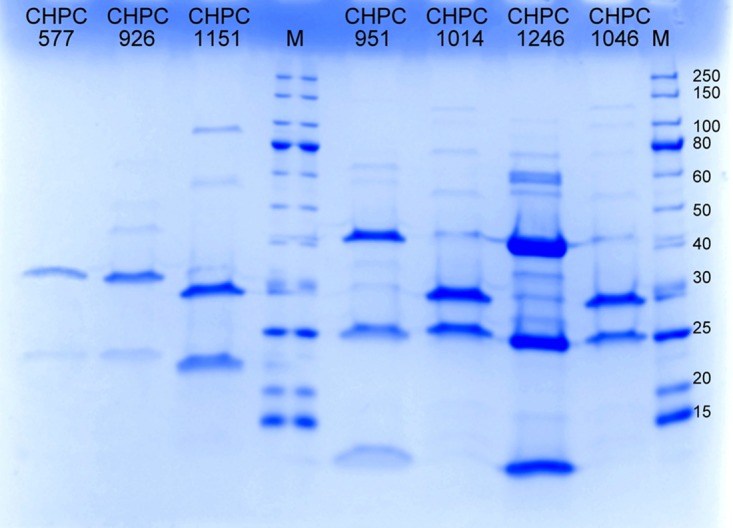
Coomassie blue-stained SDS-polyacrylamide gel showing the structural proteins of selected S. thermophilus phages from the Chr. Hansen A/S Collection (CHPC577, CHPC926, and CHPC1151, investigated phages; CHPC951 and CHPC1246, *pac*-type phages; CHPC1014 and CHPC1046, *cos*-type phages; M, marker with values in kilodaltons [New England BioLabs, USA]).

The set of main structural proteins for phages CHPC577, CHPC926, and CHPC1151 was verified by performing an SDS-PAGE analysis. According to the literature, the *pac*-type phages contain three main structural proteins of 41, 25, and 13 kDa, while the *cos*-type representatives have two main structural proteins of 32 and 26 kDa (2, 5). Two main structural proteins in phages CHPC577, CHPC926, and CHPC1151 were identified. The molecular masses of the main proteins visible on the gel were estimated to be 31 and 22 kDa for phages CHPC577 and CHPC926, and 30 and 21 kDa for phage CHPC1151 (Fig. 5).

By performing a liquid chromatography-mass spectrometry (LC-MS) assay, the bands primarily identified on the SDS-PAGE were correlated with the RASTtk annotations (14) for putative structural genes of the three phages studied (Table 2). For phages CHPC577 and CHPC926, the protein of the higher molecular weight matched the predicted major capsid protein of 31.3 kDa, encoded in both genomes by ORF 6. The protein with the lower molecular weight corresponded to the predicted major tail protein of 18.2 kDa, encoded by ORF 12 in phage CHPC926. The similar protein was not recognized for phage CHPC577 in the LC-MS analysis. The SDS-PAGE bands of phage CHPC1151 matched two proteins, the predicted major capsid protein of 30.1 kDa and the predicted tail protein of 18.6 kDa, encoded by ORFs 7 and 13, respectively.

**TABLE 2:**
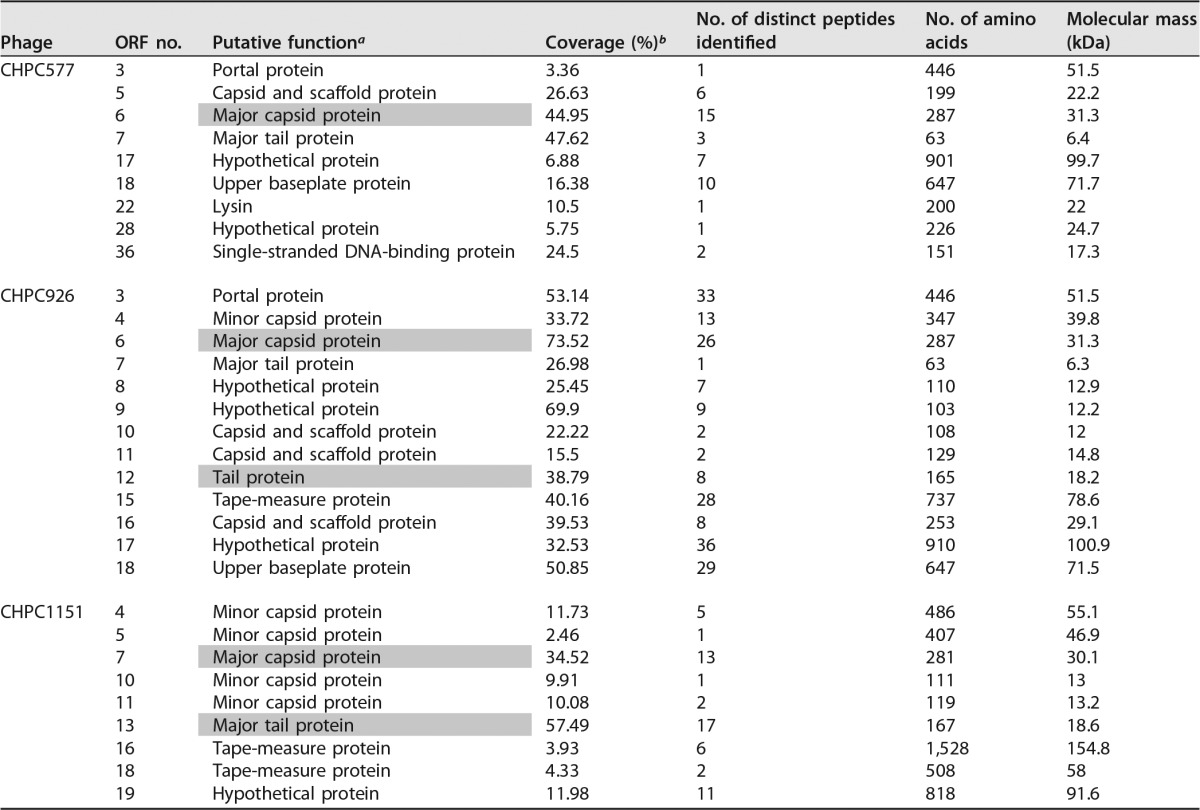
Structural proteins of phages CHPC577, CHPC926, and CHPC1151 identified by LCMS

a Proteins identified by SDS-PAGE are marked in gray.

b Coverage indicates the number of amino acids identified, expressed as a percentage of the number of amino acids in the entire protein.

Other proteins detected in the LC-MS assay for the three phages studied were assigned to the formation of a capsid, a tail protein, a tail-associated lysin, or upper baseplates on a tail tip. Additionally, proteins of unknown functions (those in ORFs 17 and 28 in CHPC577, ORFs 8, 9, and 17 in CHPC926, and ORF 19 in CHPC1151) were detected by the LC-MS analysis (Table 2).

### DNA packaging mechanism of the atypical phages.

**FIG 6:**
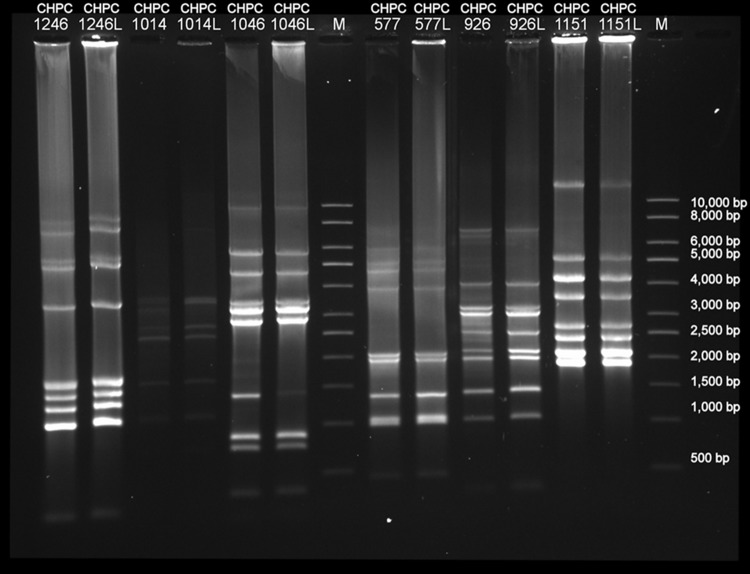
Agarose gel with enzymatic digested genomes of selected S. thermophilus phages from the Chr. Hansen A/S Collection to verify the presence of cohesive ends in the genomes (CHPC1246, *pac*-type; CHPC1014 and CHPC1046: *cos*-type; CHPC577, CHPC926, and CHPC1151, investigated phages; L, samples treated with ligase; M, 1-kb marker [Sigma-Aldrich, USA]).

The presence of cohesive ends in the DNA of phages CHPC577, CHPC926, and CHPC1151 was tested by performing genome digestion with restriction enzyme EcoRV. We did not detect the disappearance of one band or the appearance of two smaller bands when we compared the heat-treated samples with or without the addition of ligase (Fig. 6). Therefore, the presence of cohesive ends in the DNA of the three phages studied could not be confirmed, suggesting a *pac*-type packaging mechanism.

### Interactions of the similar streptococcal and lactococcal phages with S. thermophilus and L. lactis strains.

Phages CHPC577 and CHPC926 possessed structural genes homologous to the subgroup II of L. lactis P335 phages. The genome similarity included putative antireceptor-encoding genes. Therefore, we verified whether S. thermophilus and L. lactis phages could infect bacteria of both species. The host range of streptococcal phages CHPC577 and CHPC926, and lactococcal phages ul36 and P335 with their permissive hosts (CHCC2389, CHCC2138, SMQ-86, and F7/2) were checked. Additionally, two strains from the Chr. Hansen A/S Collection were chosen as negative controls: S. thermophilus CHCC9743, attacked by a phage with very low DNA homology to the tested phages, and L. lactis CHCC10675, infected by c2-type phages only (data not shown). Phages CHPC577, CHPC926, ul36, and P335 exhibited narrow host ranges. They formed visible plaques only with their original hosts and did not propagate on additional strains used in the assay (Table 3).

**TABLE 3:** Host range of the investigated S. thermophilus phages (CHPC577 and CHPC926) and L. lactis phages (ul36 and P335)

Bacterial strain^*a*^	PFU/ml of phage			
	CHPC577	CHPC926	ul36	P335
S. thermophilus CHPC2389	6 × 10^7^	Negative	Negative	Negative
S. thermophilus CHPC2138	Negative	2 × 10^9^	Negative	Negative
S. thermophilus CHPC9743 (NC)	Negative	Negative	Negative	Negative
L. lactis SMQ-86	Negative	Negative	2 × 10^10^	Negative
L. lactis F7/2	Negative	Negative	Negative	1 × 10^10^
L. lactis CHPC10675 (NC)	Negative	Negative	Negative	Negative

a NC, negative control.

Although phages CHPC577, CHPC926, ul36, and P335 did not share their hosts, they might still be able to recognize receptors on a bacterial cell wall and adsorb to them. To verify this hypothesis, adsorption tests of the four phages with the four host strains (CHCC2389, CHCC2138, SMQ-86, and F7/2) and the two negative controls (CHCC9743 and CHCC10675) were performed (Table 4). According to previous studies, a high adsorption is calculated above 70%, a low adsorption around 20 to 30%, and a very low adsorption below 10% (17, 18). All tested phages adsorbed efficiently (66 to 96%) to their permissive hosts. Additionally, a notable interaction between the examined phages and some nonpermissive hosts in the adsorption assay was detected. A high adsorption (77%) was calculated for S. thermophilus phage CHPC926 with the S. thermophilus CHCC2389 (host of CHPC577). Phage CHPC926 could not efficiently adsorb to any other nonpermissive host. On the contrary, phage CHPC577 seemed to have a broader cell wall receptor recognition. It could adsorb to S. thermophilus CHCC2138 (host of CHPC926) and L. lactis SMQ-86 (host of ul36) at the level of 25 to 27%. It seemed to attach to neither the receptors of the L. lactis F7/2 (host of P335) nor to the L. lactis and S. thermophilus negative controls. The tested L. lactis phages were more likely to recognize the receptors on the S. thermophilus strains than the nonpermissive hosts of their own species. Phages ul36 and P335 adsorbed to S. thermophilus CHCC2389 (host of CHPC577) at a level of 27%.

**TABLE 4:** Phage adsorption of the investigated S. thermophilus phages (CHPC577 and CHPC926) and L. lactis phages (ul36 and P335) to the cell wall of selected S. thermophilus and L. lactis strains

Bacterial strain	% adsorption by phage (avg ± SD)^*b*^			
	CHPC577	CHPC926	ul36	P335
S. thermophilus CHCC2389	66 ± 4	77 ± 3	27 ± 8	27 ± 4
S. thermophilus CHCC2138	25 ± 8	95 ± 1	21 ± 8	0 ± 2
S. thermophilus CHCC9743 (NC)	0 ± 16	1 ± 3	9 ± 4	7 ± 9
L. lactis SMQ-86	27 ± 14	11 ± 14	96 ± 3	11 ± 6
L. lactis F7/2	10 ± 15	11 ± 2	6 ± 3	77 ± 7
L. lactis CHCC10675 (NC)	13 ± 3	9 ± 1	19 ± 9	8 ± 15

a NC, negative control.

b Results are the average ± standard deviation from two independent experiments.

**FIG 7:**
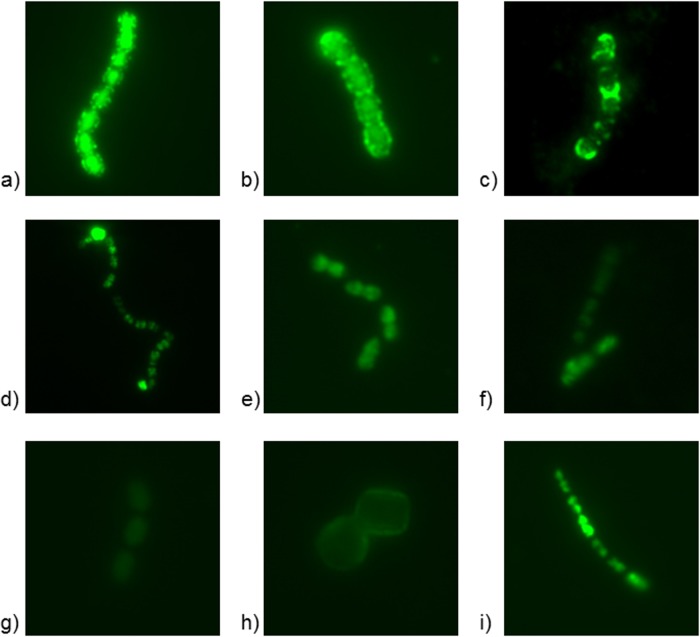
Fluorescence microscopic photographs of selected S. thermophilus and L. lactis strains and SYBR Gold DNA-stained phages taken with 1-s exposure time. The assessment of an adsorption level was correlated with the results from the adsorption test (Table 4). (a and b) High adsorption and phage DNA injection: ul36 + ul36-host (L. lactis SMQ-86) (a) and CHPC577 + CHPC577-host (S. thermophilus CHCC2389) (b). (c) High adsorption without phage DNA injection: CHPC926 + CHPC577-host (S. thermophilus CHCC2389). (d to f) Low or no adsorption: ul36 + CHPC577-host (S. thermophilus CHCC2389) (d), P335 + CHPC577-host (S. thermophilus CHCC2389) (e), and CHPC577 + CHPC926-host (S. thermophilus CHCC2138) (f). (g) No adsorption: ul36 + P335-host (L. lactis F7/2). (h) Protoplast formation: ul36 + *L. lacti*s negative control (L. lactis CHCC10675). (i) Unbound dye migration into the cell: P335 + CHPC926-host (S. thermophilus CHCC2138).

The adsorption results were verified, and the phage DNA injection was determined by taking timespan fluorescence microscopic photos of actively growing bacteria (four host strains and two negative controls) with the four DNA-stained phages (Fig. 7). In general, a clear ring-type fluorescence, characteristic of L. lactis strains with a high phage adsorption (data not shown), was not observed in the performed assay. Therefore, the mechanisms involved in the phage adsorption to the tested strains seemed to be limited by some unknown factors. Nevertheless, we observed the adsorption of phages to their permissive hosts, followed by phage DNA injection into the bacterial cells (Fig. 7a and b). The high adsorption of S. thermophilus phage CHPC926 to S. thermophilus CHCC2389 (host of CHPC577) was also noticed; however, the phage DNA was not injected into cells over time (Fig. 7c). Hence, there are additional factors related to the bacterial cell, preventing them from phage DNA injection. By analyzing the fluorescence microscopic photos, a low adsorption of S. thermophilus phage CHPC577 to the S. thermophilus CHCC2138 (host of CHPC926) could be observed (Fig. 7f), compared to cases without any adsorption (Fig. 7g). Similarly, L. lactis phages ul36 and P335 could recognize the receptors of S. thermophilus CHCC2389 (host of CHPC577) (Fig. 7d and e). Additionally, we detected protoplast formation while mixing bacteria with phage ul36, which suggested high activity of lysin present in the phage lysate (Fig. 7h). Finally, some free migration of unbound fluorescent dye into the cells was observed (Fig. 7i). The intensity of intracellular staining depended on the strain, which could imply diverse cell wall construction and permeability. Thus, it could explain a varied sensitivity of the tested bacteria toward the potential phage infection.

## DISCUSSION

We discovered three atypical phages infecting the dairy bacterium Streptococcus thermophilus, while examining a large group of phages from the Chr. Hansen A/S collection with the *pac*- and *cos*-grouping multiplex PCR method (12). Investigating morphological and genomic features of the novel phages revealed that two of them, CHPC577 and CHPC926, were closely related to a subgroup of dairy phages attacking Lactococcus lactis. The third one, CHPC1151, showed similarity with the atypical S. thermophilus phage 5093 (7).

The results of this study revealed notable genetic diversity within the S. thermophilus phage population. The three investigated representatives could not be classified into a traditional *pac* or *cos* type recognized for S. thermophilus phages (12). Additionally, they did not have the variable region VR2 recognized within their antireceptor genes (6). Therefore, we noticed the necessity for a more accurate grouping system for dairy S. thermophilus phages. Moreover, the results of this study revealed the possibility of genetic recombination of phages attacking different bacterial species, which opens new perspectives on dairy phage evolution and their adaptation strategy.

Reconsideration of the current S. thermophilus phage grouping has already been suggested when the novel phage 5093 was discovered. Although it seemed to be a *cos*-type phage with two main structural proteins, it shared more genetic similarity with *pac* group and a nondairy streptococcal prophage (7). To date, it was the only representative of this atypical species (3, 8). After discovering phage CHPC1151, a separate subgroup of S. thermophilus phages with similar characteristics could be created. Phages 5093 (7) and CHPC1151 showed homology on a genetic and morphological level as well as on the level of structural proteins. Since the presence of cohesive ends in the genome of CHPC1151 could not be confirmed, the novel phage type may comprise one representative with *pac* and another with a *cos* DNA packaging mechanism.

The characteristics of CHPC577 and CHPC926 additionally impair the accuracy of the typical classification, i.e., the *cos* type with two main structural proteins and *pac* type with three main structural proteins, commonly applied for S. thermophilus phages (5). Phages CHPC577 and CHPC926, similar to phages belonging to subgroup II of L. lactis P335 phages, seemed to use the *pac* head-full DNA packaging mechanism (13, 19–21); however, they held two main structural proteins. The genetics and morphology of phages CHPC577 and CHPC926 generate a potential for a fourth subgroup among S. thermophilus phages. The results of this study supports the very recent report presenting another group of streptococcal phages that also appears to be closely related to subgroup II of L. lactis P335 phages (22).

The genetic variability among the S. thermophilus phage population has been observed before (23). Researchers suggested that the differences within this group originated from the module exchange between various phages attacking the same species (24). Late- and early-expressed gene clusters appeared to be easily combined between S. thermophilus phages (10), which was also observed for the three atypical phages. It was noted that the lysis cassette was the only part common to all S. thermophilus phages, which confirmed previous suggestions in the literature (7, 8). Although it has been proposed that there is an efficient limit for horizontal gene transfer across bacterial species barriers in dairy phages (10), the new types of phages visibly acquired structural genetic modules from species outside the dairy S. thermophilus phage group. In phages CHPC577 and CHPC926, the late-expressed region comprised the elements homologous to phages infecting L. lactis, in phage CHPC1151, structures similar to a prophage of pathogenic Streptococcus pneumoniae (7).

The results of this study support the hypothesis that genetic exchange can either be achieved by simultaneous infection of a suitable host by two different phages or by infecting a bacterium carrying a prophage (9). The atypical phages showed homology with a prophage (as for CHPC1151) or P335-type phages that could transfer into a prophage stage (as for CHPC577 and CHPC926) (19). Moreover, cross-adsorption between similar phages infecting L. lactis and S. thermophilus, for which we found evidence, represented the first stage in the adaptation for recognizing a new host of S. thermophilus. The P335-type phages are likely to share the environment with S. thermophilus phages because, together with members of groups 936 and c2, they belong to L. lactis phages that are the most frequently isolated from dairy fermentations (16). The recombination event of phages infecting two different species could therefore occur due to the coculturing of L. lactis and S. thermophilus strains, which may take place in commercial starter cultures, often composed of both thermophilic and mesophilic LAB, used in mixtures (2, 11), or the usage of raw milk containing phages (25). The advanced domestication of two main LAB species decreases strain diversity in a dairy environment, which may result in a higher probability for generating phage recombinants with potentially expanded lytic activity. Interestingly, phages 5093 and CHPC1151 revealed homology with a prophage of S. pneumoniae (7), suggesting that the genetic recombination may also occur outside the dairy environment.

The structural modules exchanged between L. lactis and S. thermophilus phages contain genes coding for host recognition. However, we did not observe the host exchange between tested L. lactis and S. thermophilus phages. The data presented in this study confirm that S. thermophilus phages are highly host specific (2, 6, 23). There is also little or no correlation found between their DNA homology and host range (5). Nevertheless, the similarity in baseplate genes explained the adsorption to the cell wall receptors of the tested strains. Phage DNA injection and propagation on bacteria other than hosts were not observed. Hence, we support the postulate that host recognition requires some additional factors (8, 26). Such aspects are not only related to phage specificity but also to the characteristics of the host bacteria, the construction of cell wall receptors, and potential phage defense mechanisms.

In conclusion, the data provided by this study can be used for accurate characterization of dairy phages as well as for better understanding of their evolution. Further studies should consider the evidence of phages crossing bacterial species barriers when searching for a new host. More tests taking into account a wider number of both S. thermophilus and L. lactis strains and phages could be undertaken to effectively demonstrate the crossing between the two species. Analysis of bacterial genomes could bring into full view the phage-host interactions across dairy species. Identifying the genes involved in host recognition, together with the linkage between the genetic similarity of hosts and host range of phages, would be necessary steps for developing efficient strategies to combat phage attacks during large-scale dairy fermentations.

## MATERIALS AND METHODS

### Bacteria, bacteriophages, and growth conditions.

Streptococcus thermophilus and Lactococcus lactis strains and phages used for this study are listed in Table 5. Host strains were stored at −40°C in growth medium supplemented with 15% (wt/vol) glycerol. S. thermophilus strains were cultured overnight at 37°C in LM17 broth (M17 broth [Oxoid, Denmark] with 2% [wt/vol] lactose). L. lactis strains were cultured overnight at 30°C in GM17 broth (M17 broth [Oxoid] with 0.5% [wt/vol] glucose).

**TABLE 5:** List of phages and strains used in this study

Phage	Bacterial host strain	Collection or reference
CHPC577	S. thermophilus CHCC2389	Chr. Hansen A/S Collection (Hørsholm, Denmark)
CHPC926	S. thermophilus CHCC2138	Chr. Hansen A/S Collection (Hørsholm, Denmark)
CHPC1151	S. thermophilus CHCC9743	Chr. Hansen A/S Collection (Hørsholm, Denmark)
CHPC951	S. thermophilus CHCC4323	Chr. Hansen A/S Collection (Hørsholm, Denmark)
CHPC1014	S. thermophilus CHCC4327	Chr. Hansen A/S Collection (Hørsholm, Denmark)
CHPC1046	S. thermophilus CHCC7018	Chr. Hansen A/S Collection (Hørsholm, Denmark)
CHPC1246	S. thermophilus CHCC6592	Chr. Hansen A/S Collection (Hørsholm, Denmark)
	L. lactis CHCC10675	Chr. Hansen A/S Collection (Hørsholm, Denmark)
ul36	L. lactis SMQ-86	19
P335	L. lactis F7/2	13
5093	S. thermophilus CSK939	7

Phages were propagated by transferring a single plaque into 10 ml of LM17- or GM17-Ca/Mg broth (i.e., growth medium supplemented with 10 mM CaCl_2_ and 10 mM MgCl_2_) inoculated with 1% overnight culture of adequate host and incubating until full lysis had occurred. The volume was gradually brought to 20 ml by adding host culture growing in LM17- or GM17-Ca/Mg. Upon lysis, the pure phage lysates were filtered through 0.45-μm-pore-size filters (Sartorius, Germany). The propagated phages were stored at 4°C.

Phage titers as well as the host ranges of investigated phages with selected bacterial strains were determined by using the double agar overlay spot test, as described previously (27). Following overnight incubation under the appropriate growth conditions, the PFU per milliliter were calculated.

### Phage concentration.

Phages propagated in a 500-ml volume were incubated with 1 M NaCl at 4°C for 1 h and centrifuged at 13,200 × *g* for 10 min. Subsequently, 10% (wt/vol) polyethylene glycol was added to the supernatant, followed by incubation at 4°C for 2 h and centrifugation at 11,000 × *g* for 12 min. The pellet was resuspended in 7.5 ml of gelatin-free SM buffer (0.1 M NaCl, 10 mM MgSO_4_·7 H_2_O, 50 mM Tris-HCl [pH 7.5], 5 mM CaCl_2_), treated with 1 U of DNase I (Sigma-Aldrich, USA) and 1 U of RNase (Sigma-Aldrich), and incubated at 37°C for 2 h. Phages were concentrated by CsCl block gradient ultracentrifugation using a swing-out Beckman rotor 32 Ti (Beckman Coulter, USA) at 25,000 × *g* for 2 h. The concentrated phages were enumerated and stored at 4°C.

### Adsorption assays.

For the adsorption test, 10 ml of LM17 or GM17 broth was inoculated with 3% overnight culture and incubated under the appropriate growth conditions until optical density at 600 nm reached approximately 0.5. Subsequently, phage solution was added to a final concentration of approximately 10^5^ PFU/ml. A control without bacterial cells was prepared. After 15 min of incubation at 37°C (S. thermophilus) or 30°C (L. lactis), the samples were centrifuged at 15,000 × *g* for 10 min. Eight hundred microliters of the supernatant was collected. The phage concentration in the supernatant was determined using the double agar overlay spot test (27). The titer was compared to the control titer of a cell-free phage solution. The percentage of phage adsorption was calculated as [(control titer − adsorption titer)/control titer] × 100. Duplicate replications of the test were conducted.

Phage adsorption to the bacterial cell wall and DNA injection was additionally visualized under a fluorescence microscope, as described previously (28), with the modification that CsCl-concentrated phages were mixed 10:1 (vol/vol) with a 1,000-fold-diluted SYBR Gold stock solution (Invitrogen, USA). Phages were additionally immobilized by the addition of 0.1% agarose before examination with a fluorescence microscope. Photographs were taken using the Zen lite camera (Zeiss, Germany) with a 1-s exposure time. A comparison of photographs taken at different time points enabled us to define the adsorption of the phage to the cell and determine the phage DNA injection.

### Electron microscopy.

Adsorption of CsCl-concentrated phages onto freshly prepared carbon film floated from a coated mica sheet and negative staining were done as described previously (29), with the modification that 1% (wt/vol) uranyl acetate was used. The film was picked up with a 400-mesh copper grid (Agar Scientific, UK), and specimens were examined with a Tecnai 10 transmission electron microscope (FEI, The Netherlands) operated at an acceleration voltage of 80 kV.

### Identification of structural proteins.

The structural phage proteins were identified by SDS-polyacrylamide gel electrophoresis (SDS-PAGE) and liquid chromatography-mass spectrometry (LC-MS). CsCl was removed from the concentrated phages by centrifugation in two repetitions on a 100-kDa filter (EMD Millipore, USA) at 10,000 × *g* for 10 min with four volumes of SM buffer. The desalted phage particles were brought back to one volume of SM buffer and used for the assays.

Phage aliquots were mixed with two volumes of SDS-PAGE loading buffer (Laemmli sample buffer [Bio-Rad Laboratories, USA] with 5% β-mercaptoethanol) and boiled for 10 min. Proteins from a 60-μl sample were separated on the Mini-PROTEAN ready gel system (Bio-Rad Laboratories) by running electrophoresis at 200 V until the tracking dye had reached the bottom of the gel (approximately 30 min). Gels were washed with an acid solution (10% acetic acid, 40% ethanol) and stained overnight with QC colloidal Coomassie stain (Bio-Rad Laboratories).

To precipitate phage proteins for LC-MS, 75 μl of the phage solution was mixed with 75 μl of a 5% SDS solution, incubated at 80°C, and shaken at 350 rpm for 30 min. Subsequently, a 1,350-μl TCA solution (10% trichloroacetic acid, 64% [wt/vol] acetone) was added. The sample was incubated on ice for 2 h, followed by centrifugation at 10,000 × *g* at 4°C for 30 min. The supernatant was discarded, and the pellet was washed with 200 μl of 80% acetone, incubated on ice for 15 min, and centrifuged at 10,000 × *g* at 4°C for 30 min. After discarding the supernatant, the pellet was dissolved in a 20-μl urea solution (8 M urea, 50 mM Tris-HCl [pH 8.0]) and 2 μl of 450 mM dithiothreitol, followed by incubation at room temperature for 45 min. Next, 4 μl of 500 mM iodoacetamide was added, and the sample was incubated in the dark at room temperature for 60 min. After adding 74 μl of 10 mM NH_4_HCO_3_ and 2 μl of trypsin (0.1 μg/μl), the sample was incubated overnight at 37°C. The enzyme was inactivated by the addition of 5 μl of 20% trifluoroacetic acid.

The resulting peptides were analyzed by LC-MS using a Proxeon Easy-nLC HPLC (Thermo Fisher Scientific, Denmark) coupled with a Q Exactive mass spectrometer (Thermo Fisher Scientific). Ten-microliter samples were loaded on a PepMap C_18_ (3 μm, 3 Å, 75 μm by 15 cm) column (Thermo Fisher Scientific). The peptides were eluted with a chromatographic gradient ranging from 0 to 30% solvent B (100% acetonitrile, 0.1% formic acid) for 60 min at a flow rate of 100 nl/min. The mass spectrometer was operated in a data-dependent mode automatically switching between mass spectrometry (MS) and tandem MS (MS/MS). A survey MS scan (*m/z* 200 to 2,000) was acquired in the Orbitrap analyzer with a resolution of 70,000 at *m/z* 400. The top seven most intense ions were selected for MS/MS (target value, 3 × 10^6^ ions; max ion filling time, 50 ms). The peptides were subjected to higher-energy collisional dissociation (HCD) MS/MS (normalized collision energy, 28; 3,500 resolution at *m/z* 400).

The data were analyzed with Proteome Discoverer 1.4 (Thermo Fisher Scientific) using the Sequent searching algorithm against the predicted phage proteins. The MS and MS/MS results were searched with a precursor mass tolerance at 10 ppm and a MS/MS mass tolerance at 0.05 Da. The search results were filtered in Proteome Discoverer with the integrated target-decoy peptide-spectrum match (PSM) validator algorithm to a *q* value of <0.01, which ensures a PSM false-discovery rate of less than 0.01.

### Molecular DNA techniques.

Multiplex PCR differentiating between *pac*-type and *cos*-type S. thermophilus phages was performed as described previously (12). Additionally, an amplification of the DNA fragment VR2, which is known to be, with the exception of phage 5093, highly conserved for S. thermophilus phages, was performed with primers HOST1 and HOST5, as described previously (6).

To verify the presence of cohesive ends in the phage genomes, S. thermophilus phage DNA was isolated from 250 ml of phage lysate, and restriction digestion of phage DNA was performed. The Maxi lambda DNA purification kit (Qiagen, USA) was used for phage DNA purification, with the modifications described previously (30). Ten microliters of phage DNA was treated with T4 DNA ligase (New England BioLabs, USA), according to the manufacturer's protocol. The phage DNA with and without ligase treatment was digested with restriction endonuclease EcoRV (New England BioLabs) overnight at 37°C. The cut phage DNA was heat treated at 95°C for 10 min and rapidly cooled on ice. Approximately 3 μg of the products was stained with EZ-Vision Three DNA dye (Amresco LLC, USA), separated on a 0.7% agarose gel in TAE buffer (40 mM Tris-acetate, 1 mM EDTA), and visualized under UV light.

To perform full-genome sequencing, S. thermophilus phage DNA was isolated from 500 μl of phage lysate (concentration, 10^9^ to 10^10^ PFU/ml) using the procedure described previously (31), with the modifications that the phage lysate was centrifuged at 10,000 × *g* for 10 min before filtration on an ultrafiltration spin column with 0.45-μm cutoff (EMD Millipore), and the lysate with 1% SDS and 3 U/ml proteinase K (Sigma-Aldrich) was incubated at 55°C for 60 min. The DNA was purified and concentrated using a DNA Clean & Concentrator-5 kit (Zymo Research, USA), according to the manufacturer's protocol, and eluted with 8 μl of elution buffer. The DNA concentration was measured using the Qubit double-stranded DNA (dsDNA) HS assay kit (Life Technologies, USA) on a Qubit 2.0 fluorometer (Life Technologies), according to the manufacturer's protocol.

DNA sequencing libraries were prepared by using the Nextera XT DNA kit (Illumina, USA), according to the manufacturer's protocol, with 1 ng of input DNA. The final DNA concentration was measured using the Qubit dsDNA HS assay kit (Life Technologies) on a Qubit 2.0 fluorometer (Life Technologies), according to the manufacturer's protocol. The presence of individually tagged libraries was confirmed by separation on a 1% agarose gel in TAE buffer (40 mM Tris-acetate, 1 mM EDTA), staining with ethidium bromide, and visualization under UV light. The sequencing was performed using the Illumina MiSeq platform with 2 × 250-bp paired-end sequencing (Illumina).

### Sequencing data analysis.

Reads were trimmed, analyzed, and assembled using CLC Genomics Workbench 8.5 (Invitrogen, Denmark). Trimming was performed using the Trim Sequences tool. The trimmed reads were assembled using the *De Novo* Assembly tool, with the default settings, using the 64-word size. The longest contig with the highest coverage, representing the full phage genome, was used for further analysis. The genome fragments with low coverage (threshold, 100 reads) were additionally verified using PCR, followed by Sanger sequencing (Macrogen, The Netherlands).

The similarities of phage genomes with other organisms available in GenBank (http://www.ncbi.nlm.nih.gov/GenBank/), as of May 2016, were assessed by using the Basic Local Alignment Search Tool (BLAST) (32), provided by the National Center for Biotechnology Information (http://blast.ncbi.nlm.nih.gov/Blast.cgi). The assembled genomes were annotated using RASTtk (14) and verified manually by comparative genomics with the phage entries from GenBank available before June 2016. The similarity between genomes was visualized using Easyfig (15). The properties of predicted proteins were calculated by using The Sequence Manipulation Suite (33). The multiple alignment and phylogenetic tree of the investigated phages, based on the nucleotide comparison, were performed using default settings in CLC Genomics Workbench 8.5 (Invitrogen). An all-against-all protein comparison was calculated using BLAST to define homology. A homolog was considered significant if 50% of the alignment consisted of identical matches and the length of the alignment was 50% of the longer gene. The core genome of investigated phages was determined with CMG-biotools (34) using the default stringency setting.

### Accession number(s).

Whole-genome sequence data for phages CHPC577, CHPC926, and CHPC1151 are available in the GenBank database under the following accession numbers: KX879641 (CHPC577), KX879642 (CHPC926), and KX879643 (CHPC1151).

## Supplementary Material

Supplemental material
