# Supplementary material for: Novel Variants of Streptococcus thermophilus Bacteriophages Are Indicative of Genetic Recombination among Phages from Different Bacterial Species
Source: Appl Environ Microbiol. 2017 Feb 15;83(5):e02748-16. doi: 10.1128/AEM.02748-16 (PMC5311409; doi:10.1128/AEM.02748-16)
Supplement: Supplemental material [file supp_83_5_e02748-16__index.html]

Supplemental material 

# Novel Variants of Streptococcus thermophilus Bacteriophages Are Indicative of Genetic Recombination among Phages from Different Bacterial Species

## Supplemental material

- Supplemental file 1 -

  Multiple genome alignment of phages CHPC577, CHPC926, and CHPC1151 with the *S. thermophilus* phages and the subgroup II *L. lactis* P335 phages available in the NCBI database (Fig. S1); list of ORFs from phages CHPC577, CHPC926, and CHPC1151 having start codon sequences different from ATG (Table S1); features and putative functions of ORFs annotated for phages CHPC577 (Table S2), CHPC926 (Table S3), and CHPC1151 (Table S4).

  PDF, 620K
