# Supplementary material for: Novel Variants of Streptococcus thermophilus Bacteriophages Are Indicative of Genetic Recombination among Phages from Different Bacterial Species
Source: Appl Environ Microbiol. 2017 Feb 15;83(5):e02748-16. doi: 10.1128/AEM.02748-16 (PMC5311409; doi:10.1128/AEM.02748-16)
Supplement: Supplemental material [file AEM.02748-16_zam999117703s1.pdf]

Supplemental material

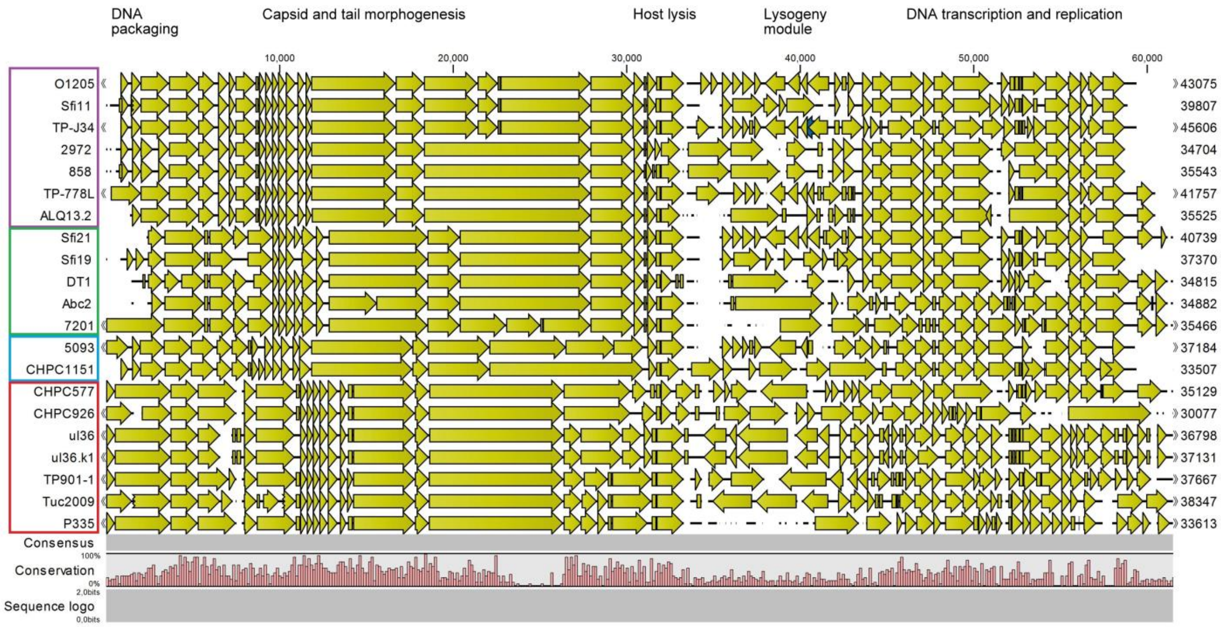

FIG S1 Multiple genome alignment of phages CHPC577, CHPC926, CHPC1151 with the *S. thermophilus* phages and the subgroup II *L. lactis* P335 phages available in the NCBI database, based on nucleotide sequences. Sequences are sorted by similarity. Four recognized homology clusters are marked in different colors.

TABLE S1 List of ORFs from phages CHPC577, CHPC926, and CHPC1151 holding start codons sequences different from ATG.

Phage	ORF no.	Starting codon
CHPC577	3, 7, 9, 38, 47	TTG
CHPC926	3, 7, 9	TTG
	1, 17	GTG
CHPC1151	2, 44	TTG
	4, 42, 45	GTG

TABLE S2 Features and putative functions of ORFs annotated for phage CHPC577.

ORF	Size [aa]	Mol mass [kDa]	pI	Putative function RASTtk	Highest identity tBLASTn	Identity [%] tBLASTn	Accession no. tBLASTn
CHPC577_01	138	15.41	9.56	hypothetical protein	*L. lactis* subsp. cremoris NZ9000	73	CP002094
CHPC577_02	463	53.16	7.20	terminase, large subunit	*L. lactis* phage ul36.k1	98	DQ394806
**CHPC577_03**	**447**	**51.54**	**4.41**	**portal protein**	*L. lactis* phage ul36.k1	93	DQ394806
CHPC577_04	347	39.80	9.29	minor capsid protein	*L. lactis* phage Tuc2009	95	AF109874
**CHPC577_05**	**200**	**22.21**	**4.64**	**capsid and scaffold protein**	*L. lactis* phage TP901-1	96	X84706
**CHPC577_06**	**288**	**31.33**	**5.83**	**major capsid protein**	*L. lactis* phage ul36	93	AF349457
**CHPC577_07**	**64**	**6.38**	**4.71**	**major tail protein**	*L. lactis* phage ul36	96	AF349457
CHPC577_08	111	12.92	4.61	hypothetical protein	*L. lactis* subsp. cremoris SK11	98	CP000425
CHPC577_09	104	12.15	4.56	hypothetical protein	*L. lactis* subsp. lactis CV56	97	CP002365
CHPC577_10	109	11.95	10.34	capsid and scaffold protein	*L. lactis* phage Tuc2009	97	AF109874
CHPC577_11	130	14.79	6.53	capsid and scaffold protein	*L. lactis* phage Tuc2009	95	AF109874
CHPC577_12	166	18.26	4.57	tail protein	*L. lactis* phage ul36	98	AF349457
CHPC577_13	117	13.46	4.70	hypothetical protein	*L. lactis* phage P335	97	DQ838728
CHPC577_14	90	10.67	10.27	hypothetical protein	*L. lactis* phage P335	99	DQ838728
CHPC577_15	917	99.20	9.88	tape-measure protein	*L. lactis* subsp. cremoris SK11	76	CP000425
CHPC577_16	254	29.12	6.81	capsid and scaffold protein	*L. lactis* subsp. cremoris SK11	94	CP000425
**CHPC577_17**	**902**	**99.82**	**5.34**	**hypothetical protein**	*L. lactis* phage ul36	72	AF349457
**CHPC577_18**	**648**	**71.70**	**5.58**	**upper baseplate protein**	*L. lactis* subsp. cremoris SK11	90	CP000425
CHPC577_19	176	19.59	9.88	serine acetyltransferase	*Lactobacillus helveticus* R0052	65	CP003799
CHPC577_20	75	8.67	6.84	holin	*L. lactis* subsp. cremoris SK11	100	CP000425
CHPC577_21	81	8.77	7.54	holin	*S. thermophilus* phage 858	91	EF529515
**CHPC577_22**	**201**	**21.99**	**4.48**	**lysin**	*S. thermophilus* phage 7201	93	AF145054
CHPC577_23	254	29.98	10.31	endonuclease	*S. thermophilus* phage S92	100	AF148563
CHPC577_24	76	8.65	3.68	lysin	*S. thermophilus* phage DT1	97	AF085222
CHPC577_25	112	12.25	10.29	hypothetical protein	*S. thermophilus* phage DT1	86	AF085222
CHPC577_26	93	10.60	10.05	hypothetical protein	*S. thermophilus* phage SK778	74	HG917970

CHPC577_27	184	21.40	8.33	hypothetical protein	*S. thermophilus* phage DT1	90	AF085222
**CHPC577_28***	**227**	**24.71**	**9.91**	**hypothetical protein**	*S. macedonicus* ACA-DC 198	45	HE613569
CHPC577_29	70	8.07	7.55	hypothetical protein	*S. thermophilus* phage phiBHN167	90	HF563658
CHPC577_30	47	5.41	10.63	hypothetical protein	*S. thermophilus* phage Abc2	88	FJ236310
CHPC577_31	43	4.98	6.49	hypothetical protein	*Streptococcus* sp. HTS5	59	CP015196
CHPC577_32	105	12.27	4.40	hypothetical protein	*S. thermophilus* phage DT1	100	AF085222
CHPC577_33	184	21.24	10.47	endonuclease	*Staphylococcus* phage StB20	64	JN700521
CHPC577_34	234	26.44	4.67	DNA-binding protein	*S. thermophilus* phage DT1	100	AF085222
CHPC577_35	444	50.43	8.06	DNA helicase	*S. thermophilus* phage ALQ13.2	99	FJ226752
**CHPC577_36**	**152**	**17.29**	**4.74**	**single-stranded DNA-binding protein**	*S. thermophilus* phage Sfi21	100	AF004379
CHPC577_37	270	30.14	7.26	replication protein	*S. thermophilus* phage Sfi21	99	AF004379
CHPC577_38	504	58.80	7.07	DNA primase/helicase	*S. thermophilus* phage SFi18	98	X77469
CHPC577_39	107	12.12	10.25	hypothetical protein	*S. thermophilus* phage 2972	93	AY699705
CHPC577_40	39	4.37	4.33	hypothetical protein	*S. thermophilus* phage TP-J34	79	HE861935
CHPC577_41	58	6.37	10.19	hypothetical protein	*S. thermophilus* phage O1205	97	U88974
CHPC577_42	55	6.65	6.52	hypothetical protein	*S. thermophilus* phage 2972	90	AY699705
CHPC577_43	63	7.17	9.45	adenine-specific methyltransferase	*S. thermophilus* phage 7201	97	AF145054
CHPC577_44	157	17.82	9.74	adenine-specific methyltransferase	*S. thermophilus* phage 7201	94	AF145054
CHPC577_45	163	19.52	4.46	hypothetical protein	*S. thermophilus* phage 5093	68	FJ965538
CHPC577_46	171	19.67	7.60	DNA-binding protein	*S. thermophilus* phage 2972	95	AY699705
CHPC577_47	272	31.83	6.30	methylase	*Streptococcus* phage phiNJ2	72	JX879087
CHPC577_48	41	4.40	6.45	hypothetical protein	*S. thermophilus* phage Abc2	95	FJ236310
CHPC577_49	236	27.79	9.60	hypothetical protein	*S. thermophilus* phage 858	87	EF529515
CHPC577_50	133	15.71	9.54	hypothetical protein	*S. thermophilus* phage ALQ13.2	82	FJ226752

Proteins identified in the LCMS are written in bold; ORFs marked in dark grey – part of the core genome of the subgroup II of *L. lactis* P335 phages; ORFs marked in light grey - part of the core genome of known *S. thermophilus* phages; * indicates ORF located on the opposite strand.

TABLE S3 Features and putative functions of ORFs annotated for phage CHPC926.

ORF	Size [aa]	Mol mass [kDa]	pI	Putative function RASTtk	Highest identity tBLASTn	Identity [%] tBLASTn	Accession no. tBLASTn
CHPC926_01	65	7.73	5.64	terminase, small subunit	*L. lactis* phage Tuc2009	98	AF109874
CHPC926_02	378	43.19	6.39	terminase, large subunit	*L. lactis* phage TP901-1	99	AF304433
**CHPC926_03**	**447**	**51.57**	**4.39**	**portal protein**	*L. lactis* phage ul36.k1	93	DQ394806
**CHPC926_04**	**348**	**39.79**	**7.93**	**minor capsid protein**	*L. lactis* phage Tuc2009	94	AF109874
CHPC926_05	200	22.21	4.64	capsid and scaffold protein	*L. lactis* phage TP901-1	96	X84706
**CHPC926_06**	**288**	**31.33**	**5.83**	**major capsid protein**	*L. lactis* phage ul36	93	AF349457
**CHPC926_07**	**64**	**6.35**	**4.71**	**major tail protein**	*L. lactis* phage ul36	98	AF349457
**CHPC926_08**	**111**	**12.89**	**4.61**	**hypothetical protein**	*L. lactis* subsp. cremoris SK11	99	CP000425
**CHPC926_09**	**104**	**12.18**	**4.56**	**hypothetical protein**	*L. lactis* phage ul36	96	AF349457
**CHPC926_10**	**109**	**11.98**	**10.34**	**capsid and scaffold protein**	*L. lactis* phage Tuc2009	96	AF109874
**CHPC926_11**	**130**	**14.79**	**6.53**	**capsid and scaffold protein**	*L. lactis* phage Tuc2009	95	AF109874
**CHPC926_12**	**166**	**18.26**	**4.57**	**tail protein**	*L. lactis* phage ul36	98	AF349457
CHPC926_13	117	13.34	4.82	hypothetical protein	*L. lactis* phage ul36	97	AF349457
CHPC926_14	90	10.73	10.27	hypothetical protein	*L. lactis* phage P335	100	DQ838728
**CHPC926_15**	**738**	**78.70**	**9.73**	**tape-measure protein**	*L. lactis* phage ul36.k1	96	DQ394806
**CHPC926_16**	**254**	**29.17**	**5.83**	**capsid and scaffold protein**	*L. lactis* subsp. cremoris SK11	94	CP000425
**CHPC926_17**	**911**	**100.96**	**5.74**	**hypothetical protein**	*L. lactis* phage P335	72	DQ838728
**CHPC926_18**	**648**	**71.59**	**5.59**	**upper baseplate protein**	*L. lactis* phage Tuc2009	90	AF109874
CHPC926_19	139	15.63	9.57	transcription termination factor	*S. salivarius* JIM8777	74	FR873482
CHPC926_20	81	8.76	9.84	holin	*S. thermophilus* phage TP-778L	88	HG380752
CHPC926_21	201	21.79	4.45	lysin	*S. thermophilus* phage S92	94	AF148563
CHPC926_22	55	6.12	10.31	endonuclease	*S. thermophilus* phage S92	100	AF148563
CHPC926_23	76	8.61	3.72	lysin	*S. thermophilus* phage ST64	100	AF148562
CHPC926_24	68	7.59	9.20	cro-like protein	*S. thermophilus* phage SK778	100	HG917969
CHPC926_25	315	36.41	4.99	hypothetical protein	*S. thermophilus* phage ALQ13.2	97	FJ226752

CHPC926_26	181	20.99	9.89	antirepressor protein	*S. thermophilus* phage 5093	95	FJ965538
CHPC926_27	70	8.23	9.42	transcription regulator	*S. thermophilus* phage ALQ13.2	100	FJ226752
CHPC926_28	47	5.48	10.41	hypothetical protein	*S. thermophilus* phage DT1	86	AF085222
CHPC926_29	288	33.37	4.69	replication initiation protein	*S. thermophilus* phage Abc2	94	FJ236310
CHPC926_30	261	30.53	9.12	DNA helicase loader	*S. thermophilus* phage 7201	94	AF145054
CHPC926_31	66	7.86	5.53	hypothetical protein	*S. thermophilus* phage Abc2	77	FJ236310
CHPC926_32	276	30.79	4.37	recombinase	*Streptococcus* phage phi30c	62	KC348599
CHPC926_33	279	32.53	6.41	hypothetical protein	*Streptococcus* phage phi5218	62	KC348600
CHPC926_34	149	16.59	5.92	single-stranded DNA-binding protein	*S. thermophilus* phage Abc2	90	FJ236310
CHPC926_35	154	18.10	10.03	holliday junction resolvase	*S. thermophilus* phage Abc2	93	FJ236310
CHPC926_36	79	9.09	9.59	hypothetical protein	*S. thermophilus* phage Abc2	89	FJ236310
CHPC926_37	58	6.52	8.48	hypothetical protein	*S. thermophilus* phage 858	91	EF529515
CHPC926_38	51	6.13	5.01	hypothetical protein	*S. thermophilus* phage DT1	93	AF085222
CHPC926_39	128	14.36	8.98	hypothetical protein	*S. thermophilus* phage Abc2	94	FJ236310
CHPC926_40	117	13.25	4.67	DNA-binding protein	*S. thermophilus* phage 5093	93	FJ965538
CHPC926_41	51	5.54	9.54	hypothetical protein	*Streptococcus* phage 20617	94	HG424323
CHPC926_42	236	27.70	9.90	hypothetical protein	*S. thermophilus* phage Sfi11	90	AF158600
CHPC926_43	150	17.33	7.90	hypothetical protein	*S. pseudopneumoniae* IS7493	51	CP002925
CHPC926_44	161	18.57	9.42	hypothetical protein	*L. lactis* phage P335	92	DQ838728

Proteins identified in the LCMS are written in bold; ORFs marked in dark grey – part of the core genome of the subgroup II of *L. lactis* P335 phages; ORFs marked in light grey - part of the core genome of known *S. thermophilus* phages.

TABLE S4 Features and putative functions of ORFs annotated for phage CHPC1151.

ORF	Size [aa]	Mol mass [kDa]	pI	Putative function RASTtk	Highest identity tBLASTn	Identity [%] tBLASTn	Accession no. tBLASTn
CHPC1151_01	139	16.24	9.02	hypothetical protein	*S. thermophilus* phage 5093	99	FJ965538
CHPC1151_02	149	16.94	6.05	terminase, small subunit	*S. thermophilus* phage 5093	100	FJ965538
CHPC1151_03	435	50.53	6.52	terminase, large subunit	*S. thermophilus* phage 5093	100	FJ965538
**CHPC1151_04**	**487**	**55.13**	**4.48**	**minor capsid protein**	*S. thermophilus* phage 5093	99	FJ965538
**CHPC1151_05**	**408**	**46.96**	**8.95**	**minor capsid protein**	*S. thermophilus* phage 5093	100	FJ965538
CHPC1151_06	206	22.94	4.65	capsid and scaffold protein	*S. thermophilus* phage 5093	100	FJ965538
**CHPC1151_07**	**282**	**30.10**	**4.63**	**major capsid protein**	*S. thermophilus* phage 5093	100	FJ965538
CHPC1151_08	62	6.82	7.54	transcription terminator	*S. thermophilus* phage 5093	100	FJ965538
CHPC1151_09	130	14.73	4.93	hypothetical protein	*S. thermophilus* phage 5093	99	FJ965538
**CHPC1151_10**	**112**	**12.96**	**8.65**	**minor capsid protein**	*S. thermophilus* phage 5093	100	FJ965538
**CHPC1151_11**	**120**	**13.19**	**10.61**	**minor capsid protein**	*S. thermophilus* phage 5093	99	FJ965538
CHPC1151_12	135	15.16	4.00	minor capsid protein	*S. thermophilus* phage 5093	100	FJ965538
**CHPC1151_13**	**168**	**18.62**	**4.59**	**major tail protein**	*S. thermophilus* phage 5093	98	FJ965538
CHPC1151_14	121	13.41	4.33	hypothetical protein	*S. thermophilus* phage 5093	100	FJ965538
CHPC1151_15	220	25.74	4.52	hypothetical protein	*S. thermophilus* phage 5093	100	FJ965538
**CHPC1151_16**	**1529**	**154.90**	**10.21**	**tape-measure protein**	*S. thermophilus* phage 5093	99	FJ965538
CHPC1151_17	240	27.99	4.93	hypothetical protein	*S. thermophilus* phage 5093	99	FJ965538
**CHPC1151_18**	**509**	**58.10**	**6.91**	**tape-measure protein**	*S. thermophilus* phage 5093	99	FJ965538
**CHPC1151_19**	**819**	**91.69**	**6.12**	**hypothetical protein**	*S. thermophilus* phage 5093	100	FJ965538
CHPC1151_20	92	10.82	7.55	holin	*S. thermophilus* phage 5093	98	FJ965538
CHPC1151_21	81	8.85	9.16	holin	*S. thermophilus* phage ALQ13.2	89	FJ226752
CHPC1151_22	201	21.86	4.60	lysin	*S. thermophilus* phage 7201	95	AF145054
CHPC1151_23	62	6.91	10.19	endonuclease	*S. thermophilus* phage S92	95	AF148563
CHPC1151_24	76	8.67	3.87	lysin	*S. thermophilus* phage 2972	100	AY699705
CHPC1151_25	68	7.64	10.27	cro-like protein	*S. thermophilus* phage DSM20617	85	HG917971
CHPC1151_26	225	26.02	7.49	hypothetical protein	*S. thermophilus* phage ALQ13.2	92	FJ226752

CHPC1151_27	70	8.07	7.55	hypothetical protein	*S. thermophilus* phage phiBHN167	90	HF563658
CHPC1151_28	249	28.83	9.79	antirepressor	*S. agalactiae* ILRI005	70	HF952105
CHPC1151_29	64	7.84	10.59	hypothetical protein	*S. thermophilus* phage 20617	72	HG424323
CHPC1151_30	292	33.83	6.19	replication initiation protein	*S. thermophilus* phage 5093	100	FJ965538
CHPC1151_31	261	30.44	8.69	DNA helicase loader	*S. thermophilus* phage 7201	97	AF145054
CHPC1151_32	61	7.22	4.50	hypothetical protein	*S. thermophilus* phage 5093	81	FJ965538
CHPC1151_33	308	35.22	4.89	phenylalanyl-tRNA synthetase	*S. thermophilus* phage K13	84	HG799496
CHPC1151_34	324	37.67	4.65	hypothetical protein	*S. thermophilus* phage 5093	82	FJ965538
CHPC1151_35	152	16.73	5.21	single-stranded DNA-binding protein	*S. thermophilus* phage Abc2	82	FJ236310
CHPC1151_36	154	18.06	9.88	holliday junction resolvase	*S. thermophilus* phage 20617	94	HG424323
CHPC1151_37	91	10.55	8.71	hypothetical protein	*S. thermophilus* phage TP-778L	88	HG380752
CHPC1151_38	57	6.38	9.11	hypothetical protein	*S. thermophilus* phage TP-J34	86	HE861935
CHPC1151_39	53	6.54	5.69	hypothetical protein	*S. thermophilus* phage 5093	96	FJ965538
CHPC1151_40	56	6.71	4.41	hypothetical protein	unknown	-	-
CHPC1151_41	38	4.45	4.37	hypothetical protein	*S. thermophilus* phage 20617	81	HG424323
CHPC1151_42	194	23.15	4.50	hypothetical protein	*S. thermophilus* phage 5093	76	FJ965538
CHPC1151_43	171	19.70	5.41	DNA-binding protein	*S. thermophilus* phage 5093	94	FJ965538
CHPC1151_44	288	31.93	8.05	DNA-cytosine methyltransferase	*S. agalactiae* 2603V/R	87	AE009948
CHPC1151_45	53	5.67	8.46	hypothetical protein	*S. thermophilus* phage Abc2	92	FJ236310
CHPC1151_46	236	27.74	9.53	hypothetical protein	*S. thermophilus* phage 7201	91	AF145054

Proteins identified in the LCMS are written in bold; ORFs marked in dark grey – the core genome of *S. thermophilus* phages 5093 and CHPC1151; ORFs marked in light grey – part of the core genome of known *S. thermophilus* phages.
